# Cohort profile: The DIabetes and ST-segment Elevation Myocardial Infarction (DISTEMI) Study

**DOI:** 10.1186/s12933-026-03097-0

**Published:** 2026-03-13

**Authors:** Clara Möser, Katsiaryna Prystupa, Martin Schön, Iryna Yurchenko, Kálmán B. Bódis, Maximilian Huttasch, Filippo Michelotti, Yuliya Kupriyanova, Vera Schrauwen-Hinderling, Cesare Granata, Gidon J. Bönhof, Alexander Strom, Christian Herder, Daniel Dörr, Sandra Trenkamp, Geronimo Heilmann, Pavel Bobrov, Klaus Straßburger, Julia Szendroedi, Mareike Cramer, Amin Polzin, Christian Jung, Malte Kelm, Volker Burkart, Robert Wagner, Michael Roden, Oana-Patricia Zaharia

**Affiliations:** 1 Department of Endocrinology and Diabetology, Medical Faculty and University Hospital Düsseldorf, Heinrich Heine University Düsseldorf, Moorenstr. 5, 40225 Düsseldorf, Germany; 2 Institute for Clinical Diabetology, German Diabetes Center, Leibniz Center for Diabetes Research at Heinrich Heine University Düsseldorf, Düsseldorf, Germany; 3 German Center for Diabetes Research (DZD), Partner Düsseldorf, Düsseldorf, Germany; 4 Department of Radiology and Nuclear Medicine, Maastricht University Medical Centre, Maastricht, The Netherlands; 5 Paul-Langerhans-Group Computational Diabetology, German Diabetes Center, Leibniz Center for Diabetes Research at Heinrich Heine University Düsseldorf, Düsseldorf, Germany; 6 Institute for Biometrics and Epidemiology, German Diabetes Center, Leibniz Center for Diabetes Research at Heinrich Heine University Düsseldorf, Düsseldorf, Germany; 7 Department of Cardiology, Pulmonology, and Vascular Medicine, Medical Faculty and University Hospital Düsseldorf, Heinrich Heine University Düsseldorf, Cardiovascular Research Institute Düsseldorf (CARID), Düsseldorf, Germany; 8 Department of Endocrinology, Diabetology, Metabolism and Clinical Chemistry (Internal Medicine 1), Heidelberg University Hospital, Heidelberg, Germany; 9 Joint Heidelberg-IDC Translational Diabetes Programm, Helmholtz Center Munich, Neuherberg, Germany

**Keywords:** Cohort profile, Myocardial infarction, Diabetes mellitus, Glucose tolerance, Insulin sensitivity, Energy metabolism, Cardiovascular function, Steatotic liver disease, Magnetic resonance imaging, Magnetic resonance spectroscopy

## Abstract

**Background:**

Humans with type 2 diabetes and/or metabolic dysfunction-associated steatotic liver disease (MASLD) are at higher risk of ST-segment elevation myocardial infarction (STEMI) and worse prognosis. However, mechanisms, prognostic factors and risk subtypes in humans with STEMI and (pre)diabetes with or without MASLD, are not fully understood.

**Methods:**

The DIabetes and ST-segment Elevation Myocardial Infarction (*DISTEMI*) study is a prospective longitudinal cohort study, recruiting humans with different degrees of glucose tolerance after recent STEMI. This cohort study has the primary objective to detect changes in glycemia and insulin sensitivity derived from the oral glucose tolerance test (OGTT) and their relationships to cardiac function. Secondary objectives address tissue-specific insulin sensitivity and organ function, focusing on adipose tissue, liver and heart. Exploratory objectives comprise multiomic analyses and measures of mitochondrial function and quality of life. At 2 and 12 months after STEMI, participants undergo comprehensive cardiometabolic phenotyping (OGTT, modified Botnia clamp-test, magnetic resonance imaging/spectroscopy/elastography, high-resolution respirometry). Magnetic resonance-based techniques are employed to assess cardiovascular function and structure, adipose tissue distribution, skeletal muscle and hepatic lipid deposition and fibrosis, and hepatic energy metabolism. Exploratory analyses include multiomics of blood, urine, and stool samples. Multiomics analyses shall allow detecting biomarkers for stratification of cardiovascular disease risk. Currently, 100 participants have been included in *DISTEMI*, of whom 29% have type 2 diabetes.

**Conclusion:**

The *DISTEMI* study integrates comprehensive cardiometabolic phenomic with multiomic profiling to identify cardiometabolic STEMI subtypes and predictors of outcomes, and to improve precision risk stratification and targeted prevention.

**Trial registration:**

NCT05046483

**Graphical Abstract:**

**Figure:**
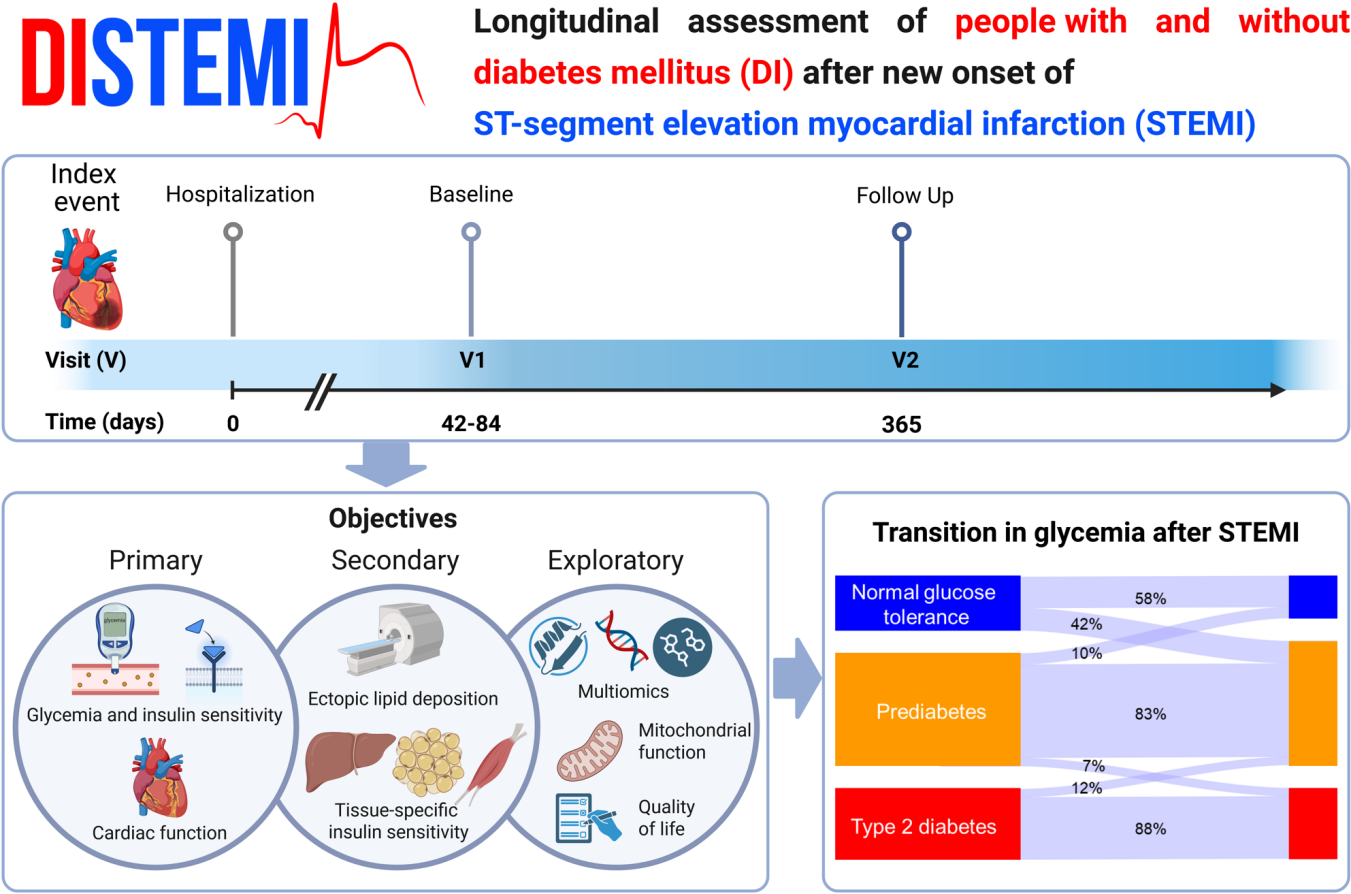

## Research insights summary

What is currently known about this topic?

1. Humans with diabetes generally have a less favorable prognosis after STEMI
2. Traditional cardiac risk factor management alone does not suffice to prevent cardiovascular risk
3. Insulin resistance is linked to cardiovascular risk but its role in STEMI outcomes remains unclear

What is the key research question?

Does insulin resistance drive adverse STEMI outcomes in humans with and without diabetes?

What is new?

1. DISTEMI addresses the impact of tissue-specific insulin sensitivity on STEMI outcomes over 1 year
2. DISTEMI shall identify longitudinal changes of cardiometabolic subtypes after STEMI
3. Multiomic analyses shall reveal non-canonical cardiometabolic risk factors of STEMI

How might this study influence clinical practice?

DISTEMI outcomes may change management after STEMI in line with precision cardiodiabetology.

## Background

Cardiovascular diseases (CVD), such as ST-segment elevation myocardial infarction (STEMI) and heart failure, are the main drivers of global mortality and morbidity in older adults [1, 2]. Type 2 diabetes and metabolic dysfunction-associated steatotic liver disease (MASLD), frequently coexist, increase cardiovascular risk and worsen outcomes of CVD [3–6], particularly with increasing liver fibrosis [7, 8]. Yet, there is an ongoing debate to which extent MASLD represents an independent risk factor or modulator of CVD morbidity and mortality [9].

Current post-STEMI risk stratification models emphasize lipid metabolism and hemodynamic load, but overlook subtle, subclinical alterations in glucose homeostasis. These alterations encompass postprandial dysglycemia, insulin resistance, beta-cell dysfunction, and low-grade (subclinical) inflammation, which are shared by states of prediabetes and overt type 2 diabetes [10–15], and modulate both myocardial injury and repair [3, 16]. Notable, an increasing proportion of STEMI occurs in the absence of established modifiable cardiovascular risk factors [17, 18], but paradoxically associates with higher early mortality risk [19].

Insulin resistance is one of the earliest abnormalities during the pathogenesis of type 2 diabetes, MASLD [10, 20], acute myocardial infarction [11, 21–23] and long-term CVD outcomes [24, 25]. In the heart, insulin resistance also associates with abnormal features of mitochondrial respiratory function and impaired myocardial substrate utilization, thereby aggravating oxidative stress and ischemia-induced myocardial injury [10, 26–29]. Nevertheless, the contribution of insulin resistance to outcomes following acute cardiovascular events such as STEMI remains insufficiently characterized [30].

Building on established subtyping of humans with prediabetes and diabetes [25, 31, 32], the DIabetes and ST-segment Elevation Myocardial Infarction (*DISTEMI*) study extends the concept of precision medicine to acute coronary syndromes. By combining cardiometabolic assessment with advanced multiomics, *DISTEMI* aims to uncover the heterogeneity in cardiometabolic alterations and identify pathways driving maladaptive remodeling and heart failure progression. By linking clinical trajectories with mechanistic insights, the *DISTEMI* study shall help to improve personalized cardiovascular medicine and outcomes after STEMI.

## Methods

### Study rationale

The objectives of DISTEMI are hierarchically structured into three objectives.

The primary objective addresses changes in glycemia (read-out: hemoglobin A1c, HbA1c) and insulin sensitivity (oral glucose insulin sensitivity index, OGIS) independently and their relationship to cardiac function (left ventricular ejection fraction, EF) as assessed by magnetic resonance imaging (MRI). OGIS is selected as the measure of insulin sensitivity due to its high feasibility across study visits, enabling adequately powered longitudinal analyses as well as accuracy for assessing insulin sensitivity as compared to the gold standard clamp-derived M-values [33].

The secondary objective is to address changes in tissue-specific insulin sensitivity (read-outs: insulin-mediated suppression of non-esterified fatty acids (NEFA) during the Botnia clamp; adipose tissue insulin resistance index (ADIPO-IR); insulin-mediated suppression of endogenous glucose production (EGP) during the Botnia clamp-test; hepatic insulin resistance index (HEP-IR)) and ectopic lipid deposition quantified by ^1^H-magnetic resonance spectroscopy (MRS).

Exploratory objectives include assessments of systemic cellular energy metabolism by leukocyte-based high-resolution respirometry, energy expenditure and substrate oxidation by indirect calorimetry, cardiorespiratory fitness by spiroergometry as well as cardiac autonomic function and measures of quality of life. Additionally, we will perform multiomics analyses integrating proteomics, transcriptomics, genomics, lipidomics and microbiome profiling. Given the high dimensionality of these data relative to sample size. These analyses are explicitly hypothesis-generating and intended to identify molecular signatures for distinguishing high-risk subgroups of STEMI for adverse outcomes, such as heart failure and major adverse cardiac events (MACE), cardiovascular death, non-fatal myocardial infarction and stroke).

### Study design

The *DISTEMI* study is a monocentric, prospective, observational cohort study comprising comprehensive functional and metabolic phenotyping as well as tissue-specific in vivo and ex vivo examinations. The study is conducted at the German Diabetes Center (Deutsches Diabetes Zentrum, DDZ), Leibniz Institute for Diabetes Research at Heinrich Heine University, Düsseldorf, Germany.

The *DISTEMI* study has been approved by the ethics committee of the Medical Faculty of Heinrich Heine University Düsseldorf (reference number: 2018-213-KFmgU, registry ID: 2018104839), registered at www.clinicaltrials.gov (identifier number: NCT05046483) and supported by the German Research Council (CRC 1116) [34]. All procedures are performed in line with the Declaration of Helsinki, current (2013) version. All participants give written informed consent to the study protocol prior to the examinations.

### Study cohort

All participants of the *DISTEMI* study were initially admitted to the Department of Cardiology, Pulmonology, and Vascular Medicine, Medical Faculty of Heinrich Heine University, University Hospital Düsseldorf, Germany, where standardized and comprehensive cardiovascular phenotyping was conducted upon hospital admission because of new onset of STEMI as part of the *SYSTEMI* cohort [35]. Through the combined framework of *DISTEMI* and *SYSTEMI* [35], developed in close collaboration with the Department of Cardiology, Pulmonology, and Vascular Medicine, we aim to map the interplay between multiorgan metabolic signatures and longitudinal outcomes after initial STEMI. The primary inclusion criterion for participation in the *DISTEMI* study is a clinical diagnosis of acute STEMI at first clinical presentation, defined according to current expert consensus [36]. All participants aged 18–80 years included in the *SYSTEMI* registry are screened and upon eligibility invited for the baseline examinations at the German Diabetes Center (DDZ).

Participants are classified as prediabetes or type 2 diabetes according to the results of a 75-g oral glucose tolerance test (OGTT) and/or HbA1c levels and further diagnostic criteria of current guidelines [37]. Remission of diabetes is defined by maintenance of HbA1c levels < 6.5% (< 48 mmol/mol) for at least 3 months in the absence of any glucose-lowering medication according to an international expert consensus statement [38].

Participants showing type 1 diabetes-related autoantibodies are excluded. Also, humans with known monogenic diabetes (e.g. maturity onset diabetes of the young), diabetes due to other causes (e.g. pancreatogenic) or gestational diabetes are not included. Table 1 summarizes the inclusion and exclusion criteria.

**Table 1:** Inclusion and exclusion criteria

Key inclusion criteria	Condition after first recent STEMI Age from ≥ 18 to ≤ 80 years Consent-able, hemodynamically stable humans, without sedation or other interfering medications
Key exclusion criteria	Poor glycemic control (HbA1c ≥ 9.0% (75 mmol/mol)) Diabetes due to other causes (e.g. pancreatogenic) Type 1 diabetes-related autoantibodies Gestational diabetes Pregnancy Acute infections/fever, infectious disease Immunosuppressive therapy Heart failure (defined as NYHA class ≥ II), renal failure (serum creatinine ≥ 1.6 mg/dl) and/or progressive liver diseases (i.e., aspartate aminotransferase or alanine aminotransferase above twice the upper limit of the reference range) Peripheral artery occlusive disease IV Severe anemia (i.e. hemoglobin <7.5 mg/dl or <10.0 mg/dl with symptoms) Severe chronic psychiatric illness or addiction Active malignant diseases Current participation in an intervention trial
Specific exclusion criteria for magnetic resonance measurements	Pregnancy Cardiac pacemaker Non-MR-compatible metallic and magnetic corpora aliena (e.g. iron splinters) or implants (e.g. mechanical heart valves, joint prostheses, cochlear implants) Waist circumference >135 cm Claustrophobia
Specific exclusion criteria for spiroergometry	ST depression or elevation reflecting acute ischemia Myocardial infarction within the last 3 months Signs of heart failure Known angina pectoris at rest or with light exertion Acute myocarditis or pericarditis Known arrhythmias Blood pressure, systolic >220 mmHg and/or diastolic >120 mmHg Known aortic stenosis, severe arteriosclerosis

Specific inclusion and exclusion criteria for participation in the “DIabetes and ST-segment Elevation Myocardial Infarction” (*DISTEMI*) study. Additionally, exclusion criteria for specific measurements in the *DISTEMI* study. STEMI, ST-segment elevation myocardial infarction; HbA1c, hemoglobin A1c; NYHA, New York Heart Association; MR, magnetic resonance

**Fig. 1:**
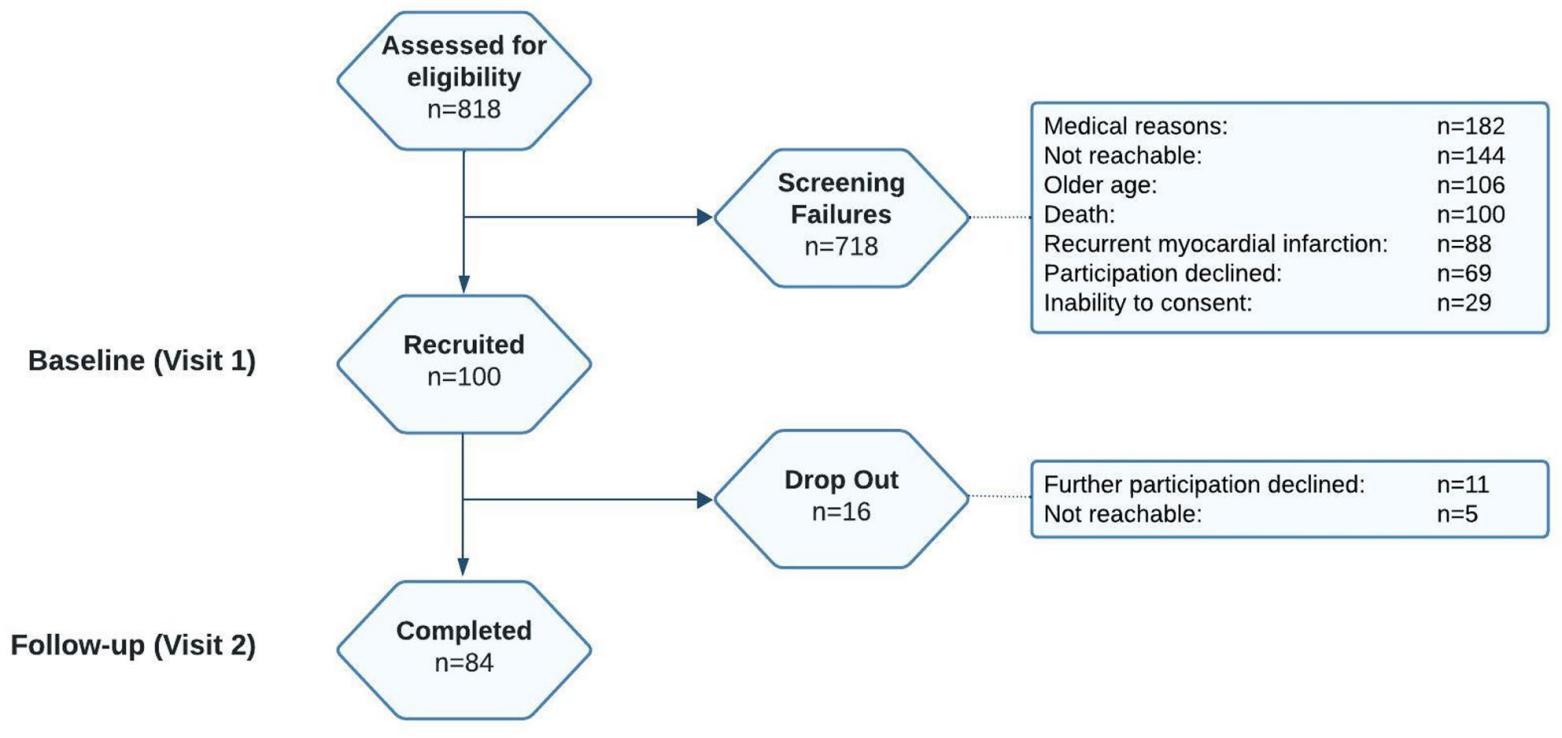
Recruitment in the *DISTEMI* study. *Legend*. Recruitment of humans with and without diabetes after recent ST-segment elevation myocardial infarction in the period between January 1st, 2019 and March 15th, 2024, hospitalized in the University Hospital of Heinrich Heine University Düsseldorf, recruited and screened for participation in the *DISTEMI* study at the German Diabetes Center in Düsseldorf. *DISTEMI*, DIabetes and ST-segment Elevation Myocardial Infarction

In January 2019 the first participant underwent the first examination (visit 1, V1). By March 2024, a total of 100 volunteers with recent STEMI have been included (Fig. 1). The attrition rate at follow-up was 16%. The recruitment rate from the *SYSTEMI* cohort is 12.2% (n = 100/818).

### Study protocol

**Fig. 2:**
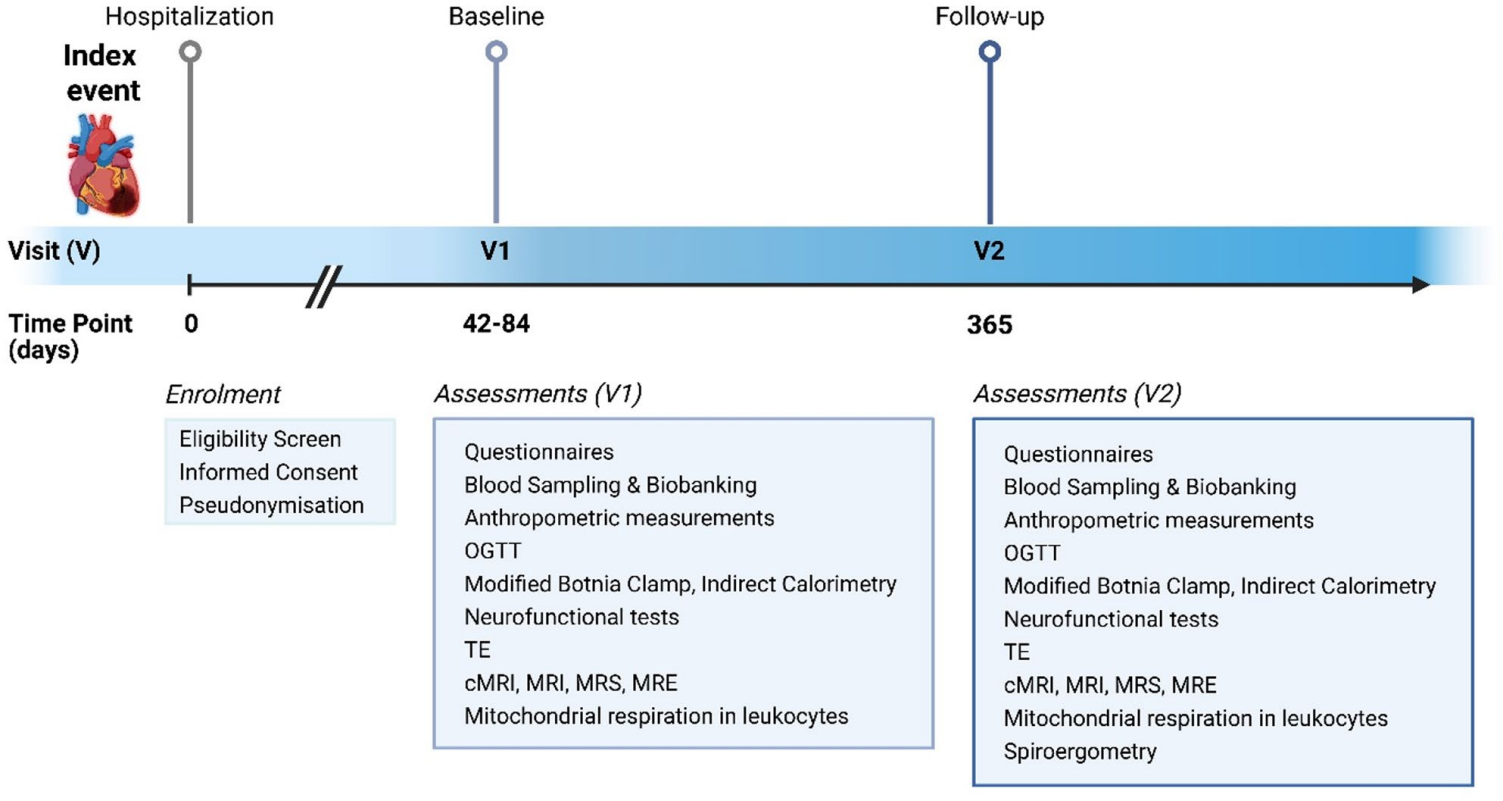
Experimental protocol in the *DISTEMI* study. *Legend*. Events and investigations within the first 365 days after recent onset of ST-segment elevation myocardial infarction (STEMI) for participants of the “Diabetes and STEMI” (*DISTEMI*) study. OGTT, oral glucose tolerance test; TE, transient elastography; (c)MRI, (cardiac) magnetic resonance imaging; MRS, magnetic resonance spectroscopy; MRE, magnetic resonance elastography; V, visit

**Fig. 3:**
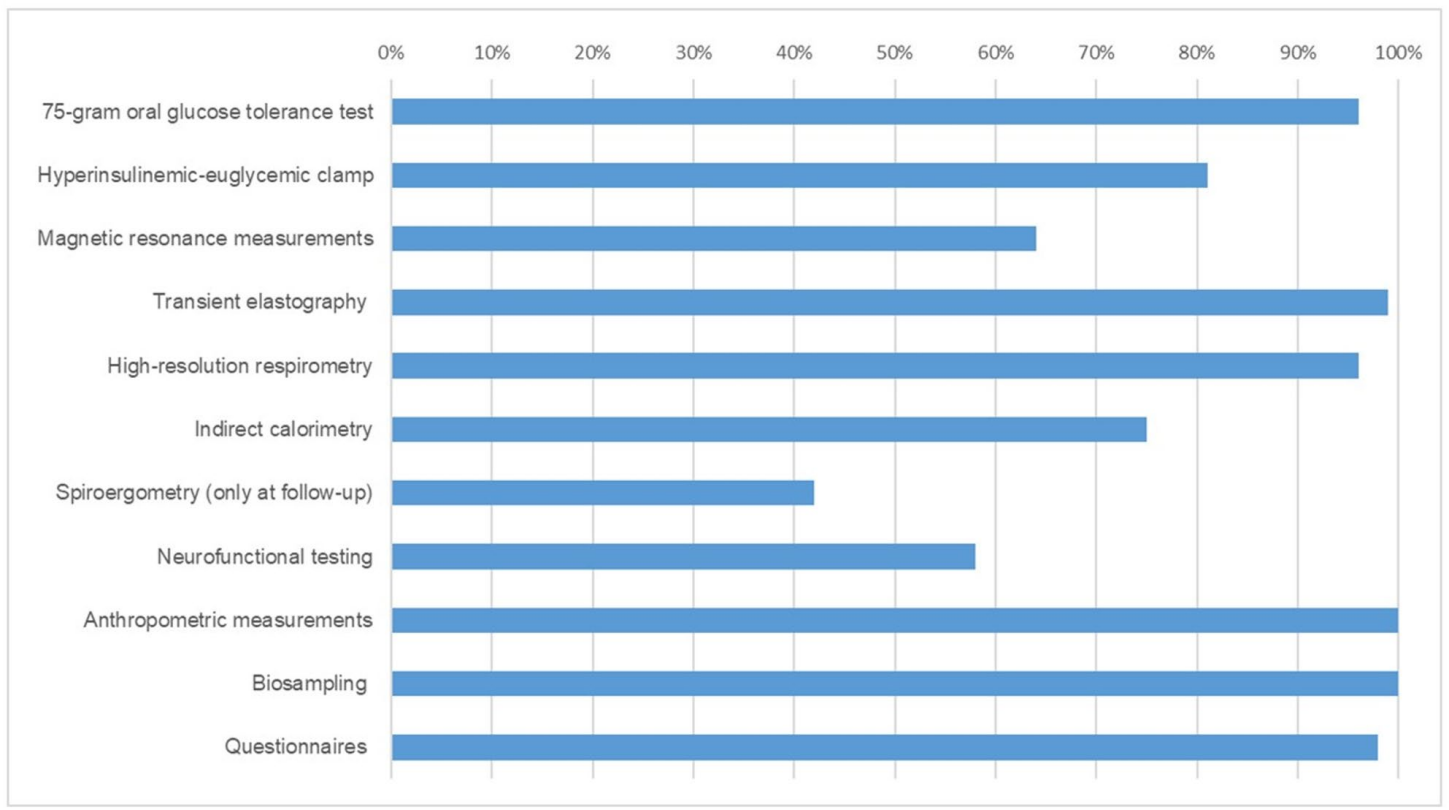
Data completeness for the assessments listed in the DISTEMI study. *Legend*. This figure represents the distribution of various measurement methods in the *DISTEMI* study, with the length of each bar indicating the percentage (0-100%) of data completeness for the assessments listed in the *DISTEMI* study protocol

Examinations are performed on 2-day visits at baseline (V1, approximately two months post-STEMI) and at 12 months after the index event (V2) (Fig. 2). On the first day, participants undergo a 75-g OGTT, neurofunctional tests, transient elastography, questionnaires and cardiac MRI. On the second day, participants undergo hepatic MRI, MRS and elastography (MRE). If participants´ history, physical examination, and laboratory results permit, they undergo the Botnia clamp-test. Completeness of the examinations is illustrated in Fig. 3.

### Experimental methods

All participants provide detailed medical history and complete questionnaires (dietary habits, physical activity, socioeconomic health, quality of life, depression, cognitive function; Table 2). They further undergo physical examination, fasting blood sampling as well as functional and metabolic tests. Table 3 shows representative baseline characteristics.

**Table 2:** Questionnaires at baseline and at 12-months follow-up

	V1	V2
Demographics		
Age, sex	✔	✘
Nationality, country of birth	✔	✘
Birth weight	✔	✘
Marital status	✔	✔
Health insurance	✔	✔
Physical disabilities	✔	✔
Diabetes history		
Time and result of oral glucose tolerance test	✔	✔
Known disease duration	✔	✔
Symptoms at time of diabetes diagnosis	✔	✔
Diabetes treatment regime	✔	✔
Diet plan and advice	✔	✔
Diabetes education	✔	✔
Family history of diabetes and other diseases	✔	✔
Myocardial infarction history		
Time of myocardial infarction	✔	✘
Time of recurrent myocardial infarction	✘	✔
Localization of myocardial infarction	✔	✔
Time of bypass or balloon dilatation, stent implantation	✔	✔
Comorbidities		
Ophthalmological	✔	✔
Hepatic	✔	✔
Renal	✔	✔
Cardiovascular	✔	✔
Neurological	✔	✔
Cerebrovascular	✔	✔
Socio-economic status		
Household composition	✔	✔
Education	✔	✔
Employment	✔	✔
Net household income	✔	✔
Personal health behavior, lifestyle		
Smoking	✔	✔
Alcohol	✔	✔
Physical activity	✔	✔
Food frequency questionnaires	✔	✔
Regular medical checks	✔	✔
Personal health history	✔	✔
Medication	✔	✔
Mental health	✔	✔
Reproductive history	✔	✔
Health-related quality of life		
WHO-5, SF-36, WHOQOL-Bref, SCL-14	✔	✔
Depression		
PHQ-D, ADS-L, PAID*	✔	✔
Informed needs, patient time		
CPS*, API*	✔	✔

Questions and questionnaires at two months (V1) and 12 months (V2) after recent myocardial infarction. ✔ stands for ‘yes’ (question asked), ✘ stands for ‘no’ (question not asked). SF-36, Short Form-36 Health Survey; WHO5, World Health Organization Five-Item Well-Being Index; WHOQOL-BREF, World Health Organization Quality Of Life (abbreviated form); SCL-14, Symptom Checklist-14; PHQ-D, Patient Health Questionnaire; ADS-L, Allgemeine Depressionsskala; PAID, problem areas in diabetes; *, only for participants with diabetes mellitus; CPS, Control Preferences Scale; API, Autonomy Preference Index

**Table 3:** Baseline characteristics in the population of the *DISTEMI* Study

	No Diabetes	Type 2 Diabetes
Numbers (n (%))	71 (71%)	29 (29%)
Age, years (n)		
18–49	10	2
50–59	18	12
60–69	31	10
70–80	12	5
Male sex (n (%))	51 (72%)	25 (86%)
BMI (kg/m^2^)	27 ± 4	30 ± 4
STEMI Localization (n)		
AWI	37	11
LWI	7	2
PWI	27	13
LWI + AWI or PWI	0	3
HbA1c (%)	5.6 ± 0.3	6.8 ± 1.1
HbA1c (mmol/mol)	38 ± 3	51 ± 12
Known diabetes duration (years)	n/a	4.1 ± 6.3
Smoking (n (%))		
Former smoker	52 (73%)	24 (83%)
Current smoker	16 (23%)	2 (7%)
Anti-hypertensive therapy (n (%))	61 (86%)	26 (90%)
Lipid-lowering therapy (n (%))	68 (96%)	29 (100%)
Anti-hyperglycemic therapy (n (%))		
Lifestyle modification	n/a	8 (28%)
Single OGLM	5 (7%)	9 (31%)
Two OGLMs	0 (0%)	10 (34%)
Insulin (with/without OGLM)	0 (0%)	2 (7%)

Data are presented as absolute numbers (n), percentages (%) or mean ± standard deviation. *DISTEMI*, humans with and without type 2 diabetes after recent ST-segment elevation myocardial infarction (STEMI); BMI, body mass index; AWI, anterior wall infarction; LWI, lateral wall infarction; PWI, posterior wall infarction; OGLM, oral glucose-lowering medication; n/a, not assessed

*Clinical phenotyping*. Body weight and height are measured by digital scale with stadiometer (SECA 764, Hamburg, Germany). Body composition (fat mass, fat-free mass) is measured using BioElectrical Impedance Analyzer System (RJL Systems, Detroit, USA) [39]. Blood pressure is measured in supine position following 30-min rest on both arms and both legs. Heart rate is recorded together with a 12-lead electrocardiogram under resting conditions. Long-term electrocardiogram (4 h) monitoring is performed to assess heart rate variability during the euglycemic state of the clamp-test.

*Glycemia and oral glucose tolerance test (OGTT)*. HbA1c represents the primary read-out of *DISTEMI*. The OGTT is the gold standard method of diagnosing prediabetes and diabetes mellitus and also permits the estimation of insulin sensitivity [37]. All participants undergo a 75-g OGTT after an overnight fasting of at least 10 h. Blood samples for measurements of plasma glucose, C-peptide and insulin are collected at timed intervals over the next three hours (0, +10, +20, +30 min followed by 30-min intervals until +180 min). The OGIS is calculated as described [33, 40].

*Modified Botnia clamp-test*. This test consists of an intravenous glucose tolerance test, for testing of beta-cell function, followed by a hyperinsulinemic-euglycemic clamp-test providing dynamic measures of insulin secretion and sensitivity [39]. The hyperinsulinemic euglycemic clamp-test has an estimated intraindividual coefficient of variation (CV) of 6% [41].

*Calculations*. Whole-body insulin sensitivity (M-value, expressed as mg*kg^−1^*min^−1^), is assessed from mean glucose infusion rates with space correction during steady state conditions of the clamp [39]. Infusion of [6,6–^2^H_2_]-glucose is used to assess hepatic insulin sensitivity by suppression of EGP and the fasting HEP-IR [42, 43]. The adipose tissue insulin sensitivity is assessed by the suppression of the plasma NEFA concentrations during the clamp-test [29, 44]. In addition, the fasting Adipo-IR is calculated as described [45]. Surrogate markers of steatosis and hepatic fibrosis are calculated from routine laboratory variables [46].

*MRI, MRS and MRE*. All magnetic resonance measurements are performed after an overnight fasting on a clinical 3-Tesla scanner (Achieva dStream X-series, Philips Healthcare, Best, The Netherlands), by trained personnel, strictly according to standardized operation procedures. Table 1 describes the specific exclusion criteria for these measurements, which can lead to missing data, for example due to claustrophobia. All data are acquired without use of contrast agents or applying a sedative. Total, subcutaneous and visceral adipose tissue volumes are quantified by whole-body MRI employing T1-weighted fast spin-echo [47] and post-processing by a trained operator using SliceOmatic® v5.0 software (Tomovision, Montréal, Quebec, Canada). Hepatic MRI, ^1^H-/^31^P-MRS and MRE are carried out for quantitative assessment of hepatic volume, lipid content, absolute concentration of ^31^P metabolites and stiffness [48–50]. Intramyocellular lipid content in tibialis anterior muscles is quantified using ^1^H-MRS [51]. In a separate session, participants undergo cardiac Cine MRI measurements (repetition time/echo time = 2.9/1.5 ms, 10 mm of slice thickness and temporal resolution of ~ 30 ms) for the characterization of left ventricular structure and function, and strains as predictors of left ventricular diastolic dysfunction and adverse outcomes [52]. The CVs of 4-10% are within the range from our previous and other studies [50, 53–55].

*Transient elastography*. Vibration-controlled transient elastography (FibroScan®, Echosens, Paris, France) is performed by a trained physician using a minimum of 10 valid measurements (interquartile range < 0.30) for the assessment of liver steatosis (controlled attenuation parameter, expressed in dB/m) [56] and liver stiffness (expressed in kPa) [57].

*High-resolution respirometry*. Leukocytes are isolated by centrifugation and are immediately analyzed for mitochondrial respiration (10^6^ per chamber). The remainder is pelleted and stored at -80 °C for subsequent analyses. Mitochondrial respiration is assessed using the high-resolution Oxygraph-2k (Oroboros Instruments, Innsbruck, Austria). Measurements are performed in duplicate using two separate substrate-uncoupler-inhibitor-titration (SUIT) protocols [58, 59]. The CVs are 5-6% (SUIT protocol 1) and 6-7% (SUIT protocol 2) and are in line with previous data [59, 60].

*Indirect calorimetry*. Indirect calorimetry is performed using the Vyntus® CPX Canopy (Vyaire, Illinois, USA) for non-invasive assessment of energy expenditure and substrate oxidation under fasted and insulin-stimulated conditions during the Botnia clamp-test [61].

*Spiroergometry*. Spiroergometry is performed to assess maximal oxygen consumption (VO_2_max) to quantify cardiorespiratory function after STEMI. The test represents an exhaustive exercise examination on a cycle ergometer (Ergometrics 900, Ergoline, Bitz, Germany) with continuous monitoring of heart rate, blood pressure, 12-lead electrocardiogram, respiratory gas exchange ratio and peak power (in Watt) [25].

*Neurofunctional tests*. Cardiovascular autonomic function tests are performed to assess cardiovascular autonomic neuropathy [62, 63]. The tests include seven indices of heart rate variability using VariaCardio TF5 (MIE Medical Research, Leeds, United Kingdom) [62, 63]. Cardiovascular autonomic neuropathy is defined by the presence of more than two abnormal quantitative autonomic function tests.

*Laboratory analyses and biosamples*. Laboratory analyses follow the national guideline for clinical laboratory analyses [64] and are regularly confirmed by ring trials (Reference Institute for Bioanalytics, Cologne, Germany). Plasma glucose, insulin, NEFA, HbA1c, circulating lipids and high-sensitive C-reactive protein are measured under fasted condition of at least 10 h and analyzed as described [39]. Type 1 diabetes-specific autoantibodies are measured as described [65]. Urine samples are collected in the morning and are processed immediately before storage at -20 °C. The biobank further contains stool samples as well as whole blood, plasma and serum samples stored at -80 °C for large-scale flow cytometry, and multiomics.

*Multiomics analyses*. Proteomics measurements based on serum/plasma samples are performed using proximity extension assay technology (Olink Proteomics, Uppsala, Sweden) [66]. The multiplex assay allows the simultaneous quantification of biomarkers including pro- and anti-inflammatory cytokines, chemokines, hormones and proteins involved in acute inflammatory and immune responses, angiogenesis, fibrosis and endothelial activation [67]*.* Lipid metabolites are analyzed using the Nightingale Health Nuclear Magnetic Resonance (NMR) spectroscopy platform (Nightingale Health Ltd, Helsinki, Finland), [68, 69]. In addition, gene expression data are obtained via mRNAseq of whole blood [70]. Multiomics analyses of stool samples are used for the characterization of host-gut microbiome interactions using deep integration of technologies including 16S rRNA sequencing, shotgun metagenomics, metatranscriptomics, metabolomics and metaproteomics.

### Statistical analyses

Data are presented as mean ± standard deviation or median (first and third quartile) for continuous variables, and as percentages (%) or absolute numbers (n) for categorical variables. Figures are created using BioRender, Lucidchart and SankeyMATIC available online. For the data management and statistical analysis, SAS 9.4 is used (SAS Institute, Cary, North Carolina, USA). Analysis of variance for generalized linear models with adjustment for age, sex, BMI, HbA1c, and for diabetes duration in type 2 diabetes is used to assess the significance of group differences or predictor effects on a read-out variable, thus accounting for potential confounding factors. Within the hierarchical framework of primary and secondary objectives, variables are analyzed within pre-specified read-out groups (e.g. glycemic, cardiac, hepatic), rather than as independent variables, to account for correlation between related measures and to limit multiplicity. For comparisons between V1 and V2 or between matched pairs, a paired Welch’s t-test is used to account for unequal variances between measurements. Based on a two-sided paired t-test at level alpha = 5% for testing differences in the hypothesis-driven primary read-out (HbA1c) between V1 and V2, a sample size of n = 87 allows to detect small to medium effect sizes (standardized mean difference) of 0.3, 0.4, 0.5 with a power of 80%, 95%, 99%. Assignment to prediabetes and diabetes subtypes is done as previously described [24, 31, 32]. Integrated multiomics, features of mitochondrial function and cardiac neurofunctional phenotyping approaches will be used in exploratory supervised machine learning algorithms to define subtypes in humans with recent STEMI and distinct outcomes. These analyses are hypothesis-generating, incorporate dimensionality reduction and regularization, and are not used as solitary variables. For this approach, global and hierarchical testing frameworks suited to integrated phenotyping studies are used [71]. Clustering will be implemented with standard safeguards against overfitting, including dimensionality reduction, regularization, and cross-validation [72].

## Discussion

The *DISTEMI* study is the first study in humans with STEMI to simultaneously and systematically assess cardiac, hepatic and glucometabolic function at defined time points within the first 12 months post-infarction across all degrees of glucose tolerance.

Despite advances in the treatment, mortality and morbidity following STEMI remain substantial, while existing risk prediction models often overlook underlying subclinical metabolic factors such as insulin resistance or ectopic lipid accumulation. Our key findings relate to the identification of distinct cardiometabolic STEMI drivers with prognostic relevance for cardiac and metabolic outcomes, including extra-cardiac comorbidities such as MASLD. Complemented by the *SYSTEMI* study dataset of inter-organ communication [35], this study shall provide unique comprehensive analyses of the systemic response to STEMI.

*DISTEMI* allows for exploring the residual metabolic heterogeneity beyond conventional cardiovascular risk factors. This is further underlined by the intensified management and participants´ high compliance as reflected by statin use in 97% and good glucometabolic control in participants with type 2 diabetes (69% with HbA1c ≤ 7%).

**Fig. 4:**
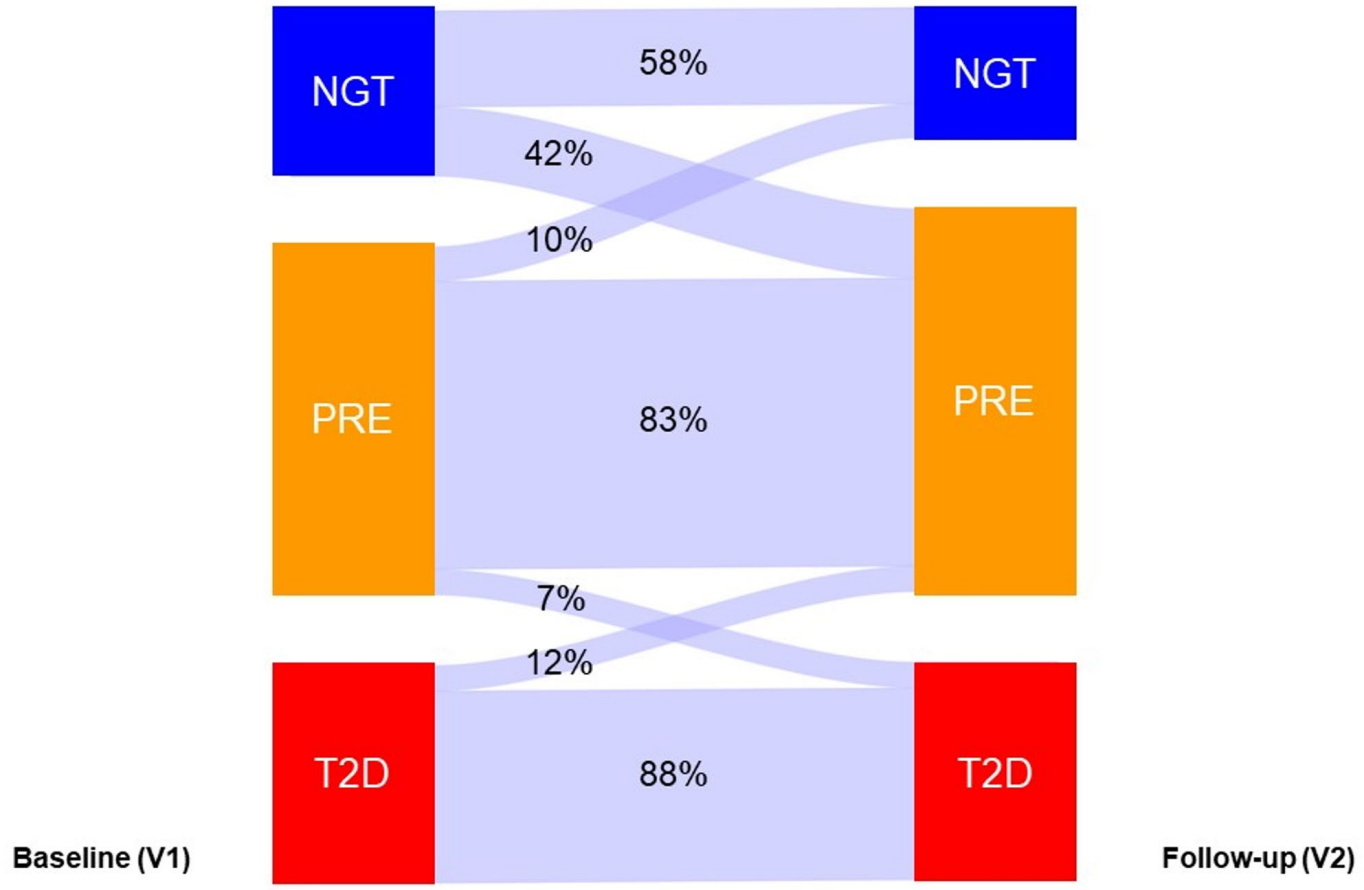
Longitudinal transitions in glucose metabolic status during the first 12 months after recent ST-segment elevation myocardial infarction. *Legend*. Glucose tolerance categories are assessed in humans with recent ST-segment elevation myocardial infarction (STEMI) at baseline (V1, 2 months post-STEMI) and at follow-up (V2, 12 months post-STEMI), participating in the *DISTEMI* study. Categories include normal glucose tolerance (NGT, blue), prediabetes (PRE, orange) and type 2 diabetes (T2D, red). Prediabetes is defined as impaired fasting glucose and/or impaired glucose tolerance and/or HbA1c between 5.7–6.4%. A subset of participants initially diagnosed with T2D achieved disease remission over time. Arrows represent the proportion of humans moving between categories over time

Altered glucose metabolism is common in humans with STEMI, shown to affect outcome-related parameters such as the left ventricular EF [73]. A recent meta-analysis showed that hyperglycemia in STEMI is strongly associated with increased short- and long-term mortality and adverse cardiovascular outcomes, with admission and fasting glucose predicting short-term risk and HbA1c better predicting long-term prognosis [74]. In our study, the distribution of glycemia with 25% normal glucose tolerance, 46% prediabetes and 29% type 2 diabetes, is similar to another contemporary cohort of humans with STEMI (35% normal glucose tolerance, 40% prediabetes and 25% diabetes) [75]. Here, we provide additional longitudinal changes in glycemia during the first 12 months after recent STEMI in *DISTEMI* (Fig. 4). Interestingly, we detected a subgroup of 12% of the cohort showing diabetes remission, possibly reflecting improvements in insulin sensitivity and beta-cell function and/or compliance with the therapeutic and preventive interventions after the acute event. In contrast, the considerable proportion of individuals developing new-onset dysglycemia at V2 (42% with new onset of prediabetes, 7% with prediabetes at V1 and new onset of type 2 diabetes) may highlight metabolic susceptibility triggered by acute cardiac injury and the subsequent systemic response, including inflammatory and other stress-related pathways.

Insulin resistance associates with multifactorial metabolic alterations such as excessive systemic NEFA and amino acid availability, abnormal energy metabolism and storage, subclinical inflammation and chronic dysglycemia [10, 76–79]. Additionally, with the presence of MASLD and its progression, CVD risk, MACE and cardiovascular mortality rise [5, 7, 8]. These maladaptive processes drive endothelial dysfunction [80] and subclinical myocardial injury, collectively accelerating disease progression and adverse cardiovascular outcomes. The *DISTEMI* study applies gold standard cardiometabolic phenotyping to provide longitudinal insights into metabolic adaptation and recovery after STEMI. Upcoming analyses will compare these data from the *DISTEMI* cohort with a cohort without CVD to distinguish infarction-related metabolic alterations from those linked to general metabolic risk. This approach may clarify tissue-specific drivers of post-infarction deterioration in glucose metabolism.

From the previously identified subtypes of pre-/diabetes, the severe insulin resistant diabetes subtype [32] and the “progressing prediabetes with fatty liver” subtype (Cluster 5) [31, 81] are associated with insulin resistance, greater hepatic lipid accumulation, and the highest CVD risk [24, 25, 31, 32, 82]. Further analyses will explore how tissue-specific insulin resistance contributes to myocardial remodeling and dysfunction following the first cardiovascular event. This concept is clinically relevant, as individualized therapies, particularly glucose-lowering treatment in humans with diabetes, have shown greater benefit in specific EF categories [83]. Sodium-glucose cotransporter-2 inhibitors and glucagon-like peptide-1 receptor agonists have demonstrated benefits on both, cardiovascular outcomes and glycemic control [84–87], highlighting the need for precision-based approaches across the cardiometabolic spectrum. There is an increasing interest in the development of clinically actionable approaches in line with the Precision Medicine in Diabetes Initiative focusing on precision diagnostics, precision prevention, precision treatment, precision prognostics, and precision monitoring [88]. Ultimately, the integration of *DISTEMI* data aims to translate advanced cardiometabolic phenotyping into novel, robust clinically actionable tools for risk stratification and targeted therapy after STEMI.

### Strengths and limitations

The main strength of *DISTEMI* is the comprehensive spectrum of cardiometabolic phenotyping combining non-invasive gold standard methods and multiomics with novel clustering approaches in humans with recent STEMI, which have not been used in combination in previous studies. The broad spectrum of structural and functional measures allows the identification, quantification and monitoring of cardiac and extra-cardiac complications and comorbidities related to (pre)diabetes within the first 12 months after STEMI. Additional data from the *SYSTEMI* study can be linked to facilitate further in-depth analyses. Distinct established laboratory parameters and non-invasive indices, as well as imaging modalities, are applied to ensure optimal non-invasive diagnostic performance for the detection of MASLD. Leukocyte-based assessments of mitochondrial respiration represents a novel, accessible technique for estimating systemic energy metabolism and may help to elucidate systemic processes triggered by recent myocardial infarction [89]. Another major strength of this study is the integration of complementary molecular data layers through multiomics experiments, including proteomics, transcriptomics, and targeted lipidomics, ensuring analytical robustness and clinical interpretability. However, the observational study design and heterogeneity of treatment, lifestyle modification, and medication could have an impact on the results, although standardized management within one center minimizes such effects [90–92]. Study participation is limited by inclusion and exclusion criteria and by the medical condition at inclusion after acute STEMI, potentially introducing selection bias towards healthier and more motivated humans. Recruitment and performance of aerosol-generating procedures (indirect calorimetry, spiroergometry, neurofunctional testing) was temporarily halted due to COVID-19-related restrictions. Furthermore, we acknowledge limitations in the robustness of statistical analyses in face of multiple read-out variables, yet a limited sample size. Finally, *DISTEMI* covers a 12-months follow-up, so that longer-term alterations cannot be monitored at present.

## Conclusion

The *DISTEMI* study shows longitudinal trajectories of glycemia after STEMI and integrates comprehensive cardiometabolic phenotyping to identify predictors of adverse outcomes in humans with recent STEMI. Additionally, multiomic profiling is explored as a tool for defining distinct cardiometabolic STEMI subtypes with differential risks for heart failure, MACE and extra-cardiac comorbidities. To this end, the *DISTEMI* study aims to advance risk stratification and guide future preventive and therapeutic strategies within the concept of precision medicine in cardiodiabetes.

## Data Availability

The datasets, generated and/or analyzed during the current study, are not publicly available since they are subject to national data protection laws and restrictions imposed by the ethics committee to ensure data privacy of the study participants. However, data are available from the authors upon reasonable request and with permission of the PI (M.R./O.P.Z.).

## Abbreviations

ADA: American Diabetes Association
Adipo-IR: Adipose tissue insulin resistance index
ADS-L: Allgemeine Depressionsskala/Center for Epidemiological Studies Depression Scale
AWI: Anterior wall infarction
API: Autonomy Preference Index
a.u.: Arbitrary units
BMI: Body mass index
CPS: Control preferences scale
CV(s): Coefficient(s) of variation
CVD: Cardiovascular disease(s)
DDG: German Diabetes Society/Deutsche Diabetes Gesellschaft
DDZ: German Diabetes Center/Deutsches Diabetes Zentrum
EF: Ejection fraction
EGP: Endogenous glucose production
HbA1c: Hemoglobin A1c
HDL: High density lipoprotein
HEP-IR: Hepatic insulin resistance index
LWI: Lateral wall infarction
LDL: Low density lipoprotein
MACE: Major adverse cardiac events
MASLD: Metabolic dysfunction-associated steatotic liver disease
MR: Magnetic resonance
MRE: Magnetic resonance elastography
(c)MRI: (Cardiac) Magnetic resonance imaging
MRS: Magnetic resonance spectroscopy
M-value: Whole-body insulin sensitivity
n/a: Not assessed
NEFA: Non-esterified fatty acids
NGT: Normal glucose tolerance
NMR: Nuclear magnetic resonance
NYHA: New York Heart Association
OGIS: Oral glucose insulin sensitivity
OGLM: Oral glucose-lowering medication
OGTT: Oral glucose tolerance test
PAID: Problem areas in diabetes
PHQ-D: Patient Health Questionnaire
PRE: Prediabetes
PWI: Posterior wall infarction
SCL-14: Symptom Checklist-14
SF-36: Short Form-36 Health Survey
STEMI: ST-segment elevation myocardial infarction
SUIT: Substrate-uncoupler-inhibitor-titration
TE: Transient elastography
T2D: Type 2 Diabetes
V: Visit
WHO5: World Health Organization Five-Item Well-Being Index
WHOQOL-BREF: World Health Organization Quality Of Life (abbreviated form)
